# First Pediatric Case of Tularemia after a Coyote Bite

**DOI:** 10.1155/2016/8095138

**Published:** 2016-01-13

**Authors:** Bruno B. Chomel, Jane A. Morton, Rickie W. Kasten, Chao-chin Chang

**Affiliations:** ^1^Department of Population Health and Reproduction, School of Veterinary Medicine, University of California, Davis, CA 95616, USA; ^2^Lucile Salter Packard Children's Hospital at Stanford, 725 Welch Road, Palo Alto, CA 94304, USA; ^3^Graduate Institute of Microbiology and Public Health, National Chung Hsing University, Taichung 402, Taiwan

## Abstract

Bite-transmitted tularemia is a rare event in humans and most of the cases have been associated with cat bites. We report the first pediatric case of tularemia caused by a coyote (*Canis latrans*) bite. Coyotes can be healthy carriers of* Francisella tularensis* and transmit this infectious agent through a bite. Pediatricians should be aware of this risk after a carnivore bite and implement appropriate antibiotic therapy, as amoxicillin/clavulanate potassium (Augmentin) may have prolonged the typical two to three days' incubation period commonly observed for tularemia after an animal bite and was not effective in preventing clinical signs in this child. Finally, it emphasizes again the importance of early and late serum samples for appropriate serodiagnostic.

## 1. Introduction

Tularemia is an acute infectious disease caused by* Francisella tularensis*, a Gram-negative bacillus, which has been reported in more than 250 animal species including mammals, birds, reptiles, fish, and invertebrates, although mammalian hosts are most commonly associated with risk of human infection, and man in North America [1]. Tularemia is principally a disease of wild lagomorphs (rabbits and hares) and rodents in the Northern Hemisphere. Tularemia is a zoonosis and humans are usually infected when handling infected animals, mainly lagomorphs or rodents [2]. Vector transmission (bites from blood sucking arthropods such as ticks, deerflies, and mosquitoes), ingestion of insufficiently cooked rabbit or hare meat, drinking contaminated water, inhalation of dust from contaminated soil, and inhalation of aerosolized bacteria are common modes of transmission [3]. Infrequently, infection is acquired following domestic or wild animal bites, mainly from domestic cats. More than 50 human cases of tularemia following cat bites have been reported between 1928 and 1993 [4]. A limited number of cases (Table 1) have been reported following bites from dogs [5], squirrels [2, 5, 6], monkeys [3, 7], a skunk [5, 8], an opossum [5], a raccoon [9], a coyote [10], a hamster [11], a prairie dog [12], and a hog or a wild boar [2, 5, 13].

The course of infection in humans, including prolonged convalescence, typically occurs over a period of 2 to 3 months. The incubation period is usually 2 to 3 days (3.3 days for 258 cases [2]) but may be less than 2 days or as long as 3 weeks.

Only two documented incidents, which occurred in Montana in 1925 and in New Mexico in 1929, respectively, have been associated with exposure to coyotes (*Canis latrans*), including one case of infection following a coyote bite [10] and one following disposal of a coyote carcass after a small cut on a finger [14]. The Montana case was in an individual who was bitten on the hand by a coyote pup when removing 5 puppies from their den [10]. Typical onset of clinical signs occurred two days after the bite incident. A persistent ulcer at the site of the bite and enlarged axillary lymph node characterized the illness. The patient developed an antibody titer of 1 : 640. The New Mexico case occurred in a patient who cut his index finger slightly with an ax and who disposed of the carcass of an adult coyote with bare hands [14]. He had killed and skinned the coyote the evening before the incident. Two days later, he complained of fever, aching body pain, and pain in the left axilla. A serum sample collected 3 weeks after the incident was positive for tularemia by agglutination at a titer of 1 : 280. The following case documents the first pediatric case of tularemia from a coyote's bite.

## 2. Case Report

We report on a 3.5-year-old Caucasian boy who was bitten by a coyote when picnicking on a summer day at Windy Hill Open Space Preserve, San Mateo County, California. Shortly before dusk, the child was attacked and bitten first on the right hand and then on the right shoulder and scalp. When brought to the Lucile Salter Packard Children's Hospital at Stanford, the head lacerations were cleaned and sutured, but the small puncture wounds on the right hand were not closed. The child was given rabies postexposure treatment, including rabies human immunoglobulins and subsequently the full series (1 mL of rabies HDCV vaccine at days 3, 7, 14, and 28). He also was started on an antibiotic treatment (Augmentin 50 mg 3 times a day (tid)). The child was followed daily by the plastic surgery department for wound care on his scalp and right hand. On the 8th day after the bite, the boy developed fever (>38.5°C) and malaise and was evaluated the following day (day 9th) for tender right axillary lymphadenopathy. On the 10th day, still on Augmentin, he was hospitalized for 24 hours. Laboratory work included a serology (slide agglutination) for* F. tularensis* and a blood culture. The child was noted to have an elevated sedimentation rate (38 mm/hr, normal range: 0–10 mm/hr) and elevated C-reactive proteins (1.3 mg/dL, normal range: 0–0.8 mg/dL). The blood culture did not yield any organism and the serology on the serum sample collected 10 days after the incident was negative for* F. tularensis* (agglutination titer < 1 : 40). On day 13 after exposure, the child developed a small papule with a small nodular pustule on the second digit proximal to the bite. On the 21st day, the child was seen again for a painful right axillary lymphadenopathy, fatigue, and a 3-day history of fever (>38.5°C). The lymph node was described as a walnut size and Augmentin was prescribed for ten days. Three days later (day 24th after the bite), the child was afebrile and playful. His right axillary lymph node, though unchanged in size, was no longer inflamed and was less tender. As indicated in the child's medical report, “in light of the good local wound healing on Augmentin therapy, the probability of staphylococcal or streptococcal infection is relatively low at this time. However, the new fever and lymphadenopathy on the same extremity than the bite are suggestive of another infectious process, likely coyote-borne, which is Augmentin resistant. Such possibility could include* F. tularensis*.” Unfortunately, because of the late suspicion of tularemia, no culture or PCR testing attempt was made from the pustule or the lymph node, given the fact that the boy had already been treated with antibiotics. The clinical symptoms seen in this child were very compatible with either tularemia or cat scratch disease. A serum sample collected approximately 2 months after the bite was negative for* Bartonella henselae* antibodies using an immunofluorescence test (IFA), but a tularemia slide agglutination test (Lot 93367LA, Difco Laboratories, Detroit, MI) revealed a* F. tularensis* titer (≥1 : 1,280) consistent with the diagnosis of tularemia. No cross-reaction was observed with* Brucella* antigen (*B. abortus* card test).

## 3. Discussion

The high antibody titer in the late serum sample confirmed the etiology of this child's infection. It is the first documented pediatric case of coyote bite-transmitted tularemia and the second ever reported case following a coyote bite [10]. Coyotes can be healthy carriers of* F. tularensis*, as they are less susceptible than rodents or lagomorphs to tularemia, as previously demonstrated [15], especially in adult coyotes compared to young coyotes [16].* Francisella tularensis* was recovered from the salivary glands of two out of three experimentally infected coyote pups [10], suggesting the possibility of human disease acquired from the bite of an infected coyote. In the present case, at least two coyotes were trapped a few days after the child's bite and they tested negative for antibodies against tularemia, plague, brucellosis, and toxoplasmosis, but positive for leptospirosis. Both coyotes also tested negative for rabies by IFA on brain tissues. Unfortunately, no attempt was made to detect* Francisella* in the oral cavity of the coyotes, because of the initial rule-out of tularemia in the boy. Because of the initial negative tularemia serology test on that child, cat scratch disease was considered. When tested serologically, both coyotes were reported to be positive for* Bartonella* spp. [17]. Further investigation revealed that coyotes are infected with a* Bartonella* species found in canids,* B. vinsonii* subsp.* berkhoffii* [17, 18]. Despite the fact that one of the two coyotes tested was likely to be the one which had bitten the child, none of them were seropositive for* F. tularensis*, therefore being healthy carriers of this bacterium. It has also been reported that tularemia is quite endemic in this part of California, where outbreaks have occurred in nonhuman primates colonies [19, 20].

Despite being uncommon, tularemia should be systematically suspected after a coyote bite, especially when the bitten person develops fever and adenopathy. This case also emphasizes the importance of early and late (>15 days) serum collection to establish an appropriate diagnosis. At the time of the first serological test, performed on a serum sample collected 10 days after the bite incident, the child had not yet mounted an elevated IgG antibody response. Furthermore, the immediate administration of antibiotics may have delayed the development of an immune response and led to a prolonged incubation period to a week instead of a 2-3 days after a bite in most documented case reports [3, 6, 10]. The treatment with Augmentin did not prevent the boy's infection but may have reduced the severity of the clinical signs by comparison with the two other documented human cases of contamination by coyotes where suppuration of lymph nodes or persistent ulceration at the bite site or infection site occurred [10, 14].

All efforts should be taken to detect or to isolate* F. tularensis* from such patients in a specialized laboratory. Serology can confirm infection retrospectively. Methods can include agglutination, ELISA, or western blotting [1]

*F. tularensis* is generally susceptible to a range of antibiotics, including fluoroquinolones, streptomycin, kanamycin, amikacin, and gentamycin and promptly treated patients have a generally favorable prognosis [1]. Tetracycline, doxycycline, and chloramphenicol may be used but are bacteriostatic and treatment must be provided for at least 14 days to prevent a relapse.

## Figures and Tables

**Table 1:** Reported cases of tularemia in humans following an animal bite (excluding cats and dogs).

Animal species	Name	Location, date	Reference(s)
Coyote	*Canis latrans*	Montana, 1925	[10]
		California, 1996	This case

Raccoon	*Procyon lotor*	Southwest-Central States 1981–1987	[5, 9]

Skunk	*Mephitis mephitis*	Greenboro, N. Carolina 1930 (reported by Francis)	[5, 8]

Opossum	*Didelphis virginiana*	N.A.	[5]

Prairie dog	*Cynomys* sp.	Spain, 2003	[12]

Ground squirrel	*Citellus richardsoni*	Montana (N.A.)	[2]

Tree squirrel	*Sciurus carolinensis*	Arkansas, 1988	[6]

Hamster	*Mesocricetus aureus*	Colorado, 2004	[11]

Squirrel monkey	*Saimiri sciurus*	California, 1970	[7]

Tamarin monkey	*Saguinus* sp.	Canada, 1978	[3]

Hog	*Sus scrofa domestica*	Iowa (N.A.)	[2, 5]

Wild boar	*Sus scrofa*	France, 1947	[13]

N.A.: not available.
